# Barriers to COVID-19 Health Products in Low-and Middle-Income Countries During the COVID-19 Pandemic: A Rapid Systematic Review and Evidence Synthesis

**DOI:** 10.3389/fpubh.2022.928065

**Published:** 2022-07-22

**Authors:** Ezekiel Boro, Beat Stoll

**Affiliations:** Faculty of Medicine, Institute of Global Health, University of Geneva, Geneva, Switzerland

**Keywords:** COVID-19, access to medicines (ATM), systematic review, low-and middle- income countries, low resource settings, global health

## Abstract

**Introduction:**

The coronavirus disease 2019 (COVID-19) pandemic has intensified the urgency in addressing pressing global health access challenges and has also laid bare the pervasive structural and systemic inequities that make certain segments of society more vulnerable to the tragic consequences of the disease. This rapid systematic review analyses the barriers to COVID-19 health products in low-and middle-income countries (LMICs). It does so from the canon of global health equity and access to medicines by proposing an access to health products in low-and middle-income countries framework and typology adapted to underscore the complex interactive and multiplicative nature and effects of barriers to health products and their root cause as they coexist across different levels of society in LMICs.

**Methods:**

Modified versions of the Joanna Briggs Institute (JBI) reviewers' manual for evidence synthesis of systematic reviews and the PRISMA-ScR framework were used to guide the search strategy, identification, and screening of biomedical, social science, and gray literature published in English between 1 January 2020 and 30 April 2021.

**Results:**

The initial search resulted in 5,956 articles, with 72 articles included in this review after screening protocol and inclusion criteria were applied. Thirty one percent of the articles focused on Africa. The review revealed that barriers to COVID-19 health products were commonly caused by market forces (64%), the unavailability (53%), inaccessibility (42%), and unaffordability (35%), of the products, incongruent donors' agenda and funding (33%) and unreliable health and supply systems (28%). They commonly existed at the international and regional (79%), health sectoral (46%), and national cross-sectoral [public policy] (19%) levels. The historical heritage of colonialism in LMICs was a commonly attributed root cause of the barriers to COVID-19 health products in developing countries.

**Conclusion:**

This review has outlined and elaborated on the various barriers to health products that must be comprehensively addressed to mount a successful global, regional, national and subnational response to present and future epidemics and pandemics in LMICs.

## Introduction

The coronavirus disease 2019 or COVID-19 was officially declared a Public Health Emergency of International Concern (PHEIC) and a pandemic by the WHO on 30 January and 11 March 2020, respectively (1). COVID-19 is caused by an infection with the severe acute respiratory syndrome coronavirus 2 (SARS-CoV-2), a novel strain that is believed to have originated from Huanan market in Wuhan, capital city of Hubei Province in the People's Republic of China (2). COVID-19 was first globally identified on 31 December 2019 by the WHO after media reports started to circulate about a cluster of cases of atypical viral “pneumonia of unknown origin” in China (1, 2). As of 5 June 2022, there have been over 531 million cases and 6.3 million deaths of COVID-19 in more than 185 countries and territories worldwide (3). COVID-19 is still officially declared by the WHO as a PHEIC and a pandemic as at the time of writing.

This unprecedented health crisis has intensified the urgency in addressing some of the pressing global health access challenges that must be surmounted for us to live in a truly equitable world. It has particularly revealed the weaknesses of the current global governance structures and mechanisms for preparing and responding to health crises—especially those caused by emerging and re-emerging infectious diseases—and it has shown how health is inextricably linked to other aspects of societal and economic growth and development globally. COVID-19 has also exposed the inequities of global public health access to health products all over the world. In the wake of the pandemic in 2020, there were acute shortages and a lack of access to life saving medical equipment like ventilators, and other personal protective equipment (PPE) like face masks, face shields, hazmat suits, and so forth in HICs and LMICs alike (4, 5). While access to many of these health technologies has significantly improved in all high-income countries, many low-and middle income countries still struggle with access to COVID-19 health products.

COVID-19 has also laid bare the pervasive structural and systemic inequities that make certain constituencies of society more vulnerable to the tragic consequences of the disease. For instance, we have clearly seen the “weathering” effects of systemic racism and structural marginalization that has made BIPOC—who are disproportionately more likely to have underlying health conditions like high blood pressure, diabetes, heart disease and kidney failure—suffer higher morbidity and mortality compared to their white counterparts (6–8). These structural inequities are also reflected in other forms of structural discrimination and supremacy such as ableism, ageism, gender discrimination and the effects of capitalism, classism and populism that typify the distribution of COVID-19 morbidity and mortality, in high-income, middle-income and low-income countries of the world (9–13). Several public health measures/non-pharmaceutical interventions (like regular hand washing, wearing of face masks, physical distancing, shelter in place and (semi) lockdown restrictions) have been globally recommended and implemented to mitigate the risks and spread of SARS-CoV-2 among and across populations around the world (14–16). These measures (though arguably theoretically sound), have also been impractical for various segments of populations across the world, fundamentally due to access inequities in various settings from a lack of adequate housing conditions and facilities or residence in overcrowded slums/housing shelters, poor access to power supply and electricity, to a lack of clean water or hand sanitizers and poor access to safe and improved WASH facilities (17–22). The health and economic consequences of COVID-19 have also exacerbated food insecurity and inequities in access to education and finance globally, especially in resource-constrained settings (23–26).

The COVID-19 health crisis—despite its devastating health and economic consequences—presents a unique opportunity for global public health leaders, policy makers and practitioners to address the various access inequities it has highlighted, especially regarding access to COVID-19 health tools in low-resource settings. A preliminary search for scoping or systematic reviews on “barriers to COVID-19 health products in low-and middle-income countries” was conducted in the PubMed, Cochrane and Google Scholar electronic bibliographic databases in April 2021 (More details in Supplementary Table 3). The search did not yield any results and so therefore, this is the first review of the existing evidence on barriers to COVID-19 health products in low-resource settings, to the authors' best knowledge. Examining the evidence is critical if we are to tackle these issues, hence the need for a rapid systematic review of the literature. Rapid systematic or scoping reviews are useful in global health to summarily identify and map the available evidence on certain global health topics or issues and they can help inform policy making or decision making and practice and reveal existing knowledge gaps for future research (27–31).

The aim of this rapid review was to map and summarily synthesize the available evidence from secondary literature sources on COVID-19 by examining the barriers to health products in low-and middle-income countries underscored by the health crisis. We also further examined the root cause of these barriers based on the literature gathered. The research question for this study was: “what are the barriers to COVID-19 health products for people living in low-and middle-income countries during the COVID-19 pandemic?” This review will (a) inform global public health leaders, policy makers and practitioners about the various barriers to COVID-19 health products in resource-constrained settings and the root cause of these barriers, (b) aid researchers in understanding the knowledge gaps that still exist on this issue to guide further research.

**Figure 1:**
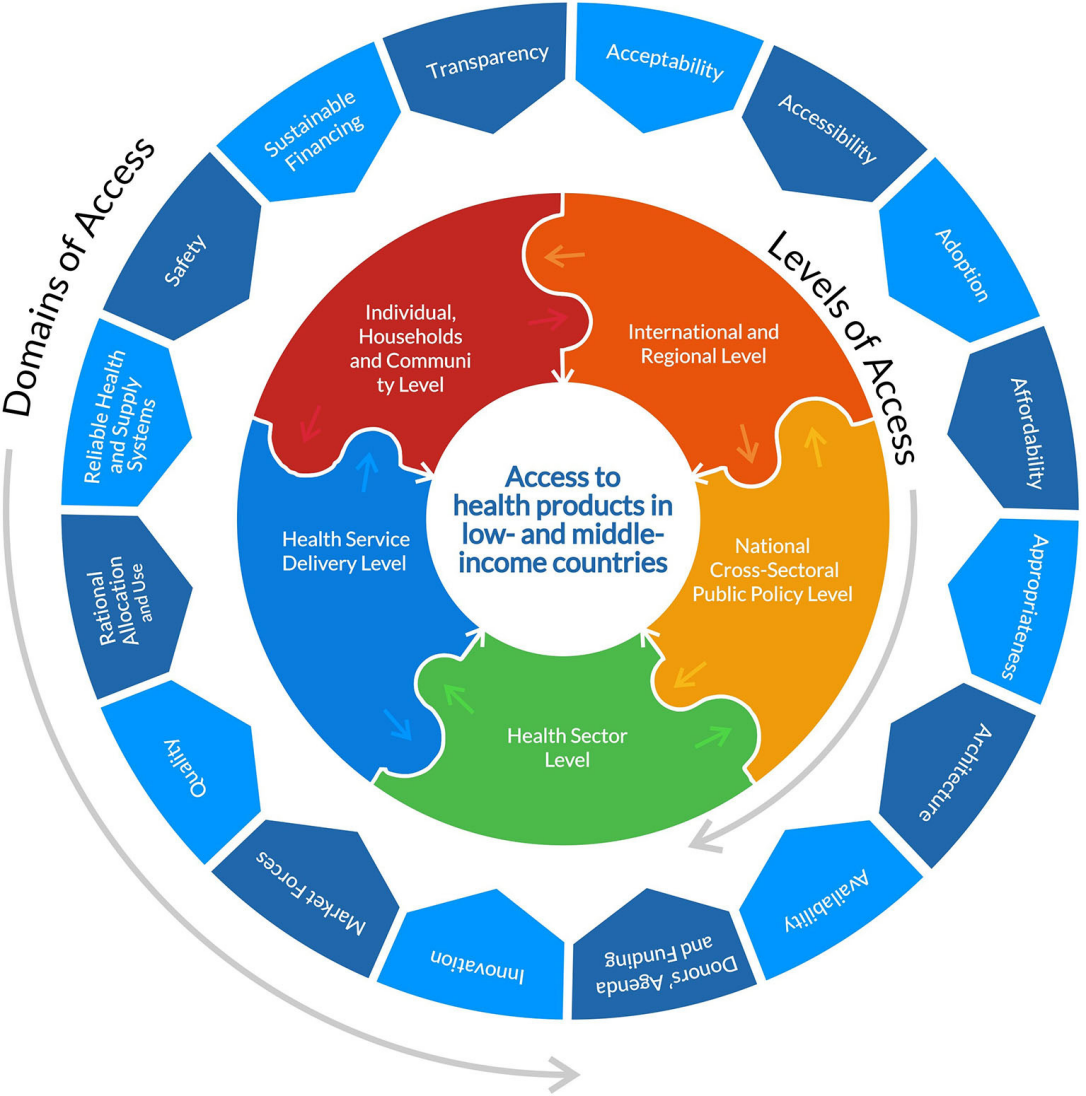
Diagram showing the domains and levels of access adapted from Bigdeli et al. (33) and the JBI model of evidence based healthcare (https://jbi.global/jbi-model-of-EBHC).

This rapid review will analyse the barriers to COVID-19 health products from the canon of global health equity and access to medicines, as elaborated by Wernli et al. (32) and WHO's Bigdeli et al. (33). Global health according to Wernli et al. (32) is “a *system-based, ecological and transdisciplinary approach which seeks to provide innovative, integrated and sustainable solutions to address complex health problems across national boundaries, and improve health for all*.” We propose in this paper, an access to health products in low-and middle-income countries framework and typology adapted from Bigdeli et al. (33) of five levels and sixteen domains of access that determine barriers to health products in low-resource settings. The levels of access are (i) international & regional level, (ii) national cross-sectoral public policy level, (iii) health sector level, (iv) health service delivery level and (v) individual, households and community level. The domains^1^ of access are (i) acceptability, (ii) accessibility, (iii) adoption, (iv) affordability, (v) appropriateness, (vi) architecture, (vii) availability, (viii) donors' agenda & funding, (ix) innovation, (x) market forces, (xi) quality, (xii) rational selection, (xiii) reliable health & supply systems, (xiv) safety, (xv) sustainable financing and xvi) transparency (Please find the framework and typology of the various levels and domains in Table 1; Figure 1). This adapted framework and typology will help to better understand the complex interactive and multiplicative nature and effects of barriers to health products and their root cause as they coexist across different levels of society. WHO recommends a holistic health systems strengthening approach that addresses these domains of barriers to health products as they occur across the various levels of the global public health systems in LMICs.

**Table 1:** Framework & typology of levels and domains of access adapted from Bigdeli et al. (33).

	**Description**
**Levels of access**	
International and regional level	Encompasses the global health governance, global governance for health and governance for global health institutions and processes that have a direct or indirect impact on access to health products globally (34).
National cross-sectoral public policy level	Comprises all non-health ministerial or sectoral public policy institutions and processes that directly or indirectly determine in-country access to health products.
Health sector level	Includes the various components of the health system that shape health and health product policies in a nation state (35).
Health service delivery level	Covers all formal and informal, private and public stakeholders, processes and activities that operationalize access to health products sub-nationally, regionally or locally in-country.
Individual, households and community level	Comprises all individuals, households and communities in various socio cultural contexts and the behaviors, attributes and other determinants that influence their uptake of health services and access to health products at the point of use.
**Domains of access**	
Acceptability	Refers to the characteristics of health products and health services that appeal to the end-users and the attitudes and expectations that will determine their consumption.
Accessibility	Refers to the geographical circumstances and contexts that determine access to health products and health services on the demand and supply side.
Adoption	Refers to demand for health products at all the levels of access described above.
Affordability	Refers to the prices of health products and health services and the financial circumstances or contexts that determine an end users' uptake and/or a payor/payee's ability and willingness to pay.
Appropriateness	Refers to the unique attributes of health products and other health interventions that reflect the environmental, socio-economic and cultural realities of the people who will use them and settings where they will be used.
Architecture	Refers to the organizational relationships (including collaborations and partnerships) or coherence of governance frameworks at the local, national, regional and international levels that determine access to health products (36).
Availability	Refers to how obtainable health products are by quantity and type on the demand and supply side (including by manufacturing, forecasting, procurement, distribution and delivery functions).
Donors' agenda and funding	Refers to foreign policy, geopolitical and other external objectives, goals and commitments that determine development assistance/aid and/or influence national health plans or policies for access to health products in recipient countries (37).
Innovation	Refers to R&D efforts and activities that lead to discoveries and development of new health products, or new delivery channels/platforms or new formulations/indications for old health interventions or incremental solutions such as simplification of therapeutic dosage and packaging, including for combination therapies.
Market forces	Refers to the economic dynamics of demand and supply in formal and informal markets that commodifies medicines and other health products to determine its value and use at all access levels.
Quality	Refers to objective and/or subjective standards of measure that underpin the value ascribed to health products.
Rational allocation and use	Refers to the efficient or equitable allocation and use of health products by rationalizing medical technological distribution, use and choices across all access levels.
Reliable health and supply systems	Refers to the optimal functionality of health and supply systems across all access levels that ascribes and asserts trust in the value and use of health products and health services.
Safety	Refers to the freedom from deleterious risks and harm after medicines and other health products have been thoroughly assessed pharmacologically or clinically and regulatorily approved for use or consumption.
Sustainable financing	Refers to the long term resource mobilization for access to health products across all access levels.
Transparency	Refers to the openness of relevant information such as manufacturing costs, research and development investments, technology know-how, clinical data, etc that influence the purchase of health interventions for payors and payees across all access levels (38).

Finally, health products as used in this review refers to all health commodities, tools and/or technologies that are critical for the healthcare response to COVID-19 including but not limited to diagnostics, PPE, therapeutics and vaccines. Also, low- and middle-income countries is a term used to collectively describe low-income, lower-middle income and upper-middle income countries with a gross national income per capita of ≤ $12, 055 USD as defined by the World Bank in 2018 using the Atlas method.

## Methods

### Study Design

This research study was conducted using a modified version of the Joanna Briggs Institute (JBI) reviewers' manual for evidence synthesis of scoping reviews (which is based on Arksey and O'Malley's and Levac et al.'s earlier scoping review frameworks) as a guide to establish an a priori protocol for the search strategy, eligibility criteria and other assumptions used for this review (39–41). The JBI framework includes the following steps: (1) define the research question(s), (2) identify relevant literature, (3) screen and select literature, (4) extract and/or chart relevant data from the literature, (5) collate/summarize/report the findings. A modified version of the PRISMA-ScR (Preferred Reporting Items for Systematic Reviews and Meta-Analyses extension for Scoping Reviews) framework was also used as a guide to identify and screen the literature search results (see Figure 1).

### Literature Search Strategy

An initial search of PubMed and Google search engine was conducted to generate relevant key words, index terms and a search string for the review. A comprehensive search strategy was used to perform advanced bibliographic searches of scientific electronic databases (in the biomedical and social sciences) using a standard set of key words and search strings (More details in Supplementary Table 4). The search strategy also included a review of reference lists from selected publications and purposive searches of key journals.

### Data Sources

This review included literature that discusses or sheds a light on the barriers [or root cause(s) of global health access] to COVID-19 health products for people living in LMICs. A PCC (Population, Concept and Context) criteria checklist was developed to ensure compliance in terms of the population, contexts and concepts elaborated in the papers that were used for the review (More details in Supplementary Table 5). Articles published in English language between 1 January, 2020 and 30 April, 2021 were sourced from scientific and gray literature sources in various (research) formats. The PubMed, Web of Science, Embase and PhilPapers databases were searched and original empirical studies, reviews, commentaries, (opinion) editorials and correspondences included. Publications by the WHO, World Bank and New York Times were also purposively searched and reviewed. All papers were independently identified and screened by the authors using keywords, title, abstract, and full text to determine if they met the inclusion and exclusion criteria and papers that did not meet the eligibility criteria were excluded. The Zotero citation software was used to collate and manage imported references. This research study did not need to seek or receive ethical approval from any research ethics review board.

### Data Extraction

The following information was extracted from each study: (i) author(s), (ii) date of publication, (iii) article type, (iv) research design/methodology, (v) level of evidence for meaningfulness using a modified version of JBI's evidence grade outline, (vi) domain(s) of access as barrier(s) (vii) level(s) of barrier(s), (viii) research context(s)/setting(s) described (see Table 2 for details). The data was organized into tables using Microsoft® Excel® for Microsoft 365 MSO (Version 2203 Build 16.0.15028.20178) 64-bit. Synthesis and analysis were guided by important themes from the reviewed literature.

**Table 2:** Data extraction and review summary.

**S/N**	**Author(s)**	**Date of publication**	**Article type**	**Research design/** **methodology**	**Modified level of evidence for meaningfulness^**a**^**	**Domain(s) of access as barriers**	**Level(s) of barrier(s)**	**Research context(s)/setting(s)**
1	Hopman et al. (42)	16 March, 2020	Commentary	N/A	5 (Expert opinion)	Affordability; accessibility; market forces	Health sector; International & regional	LMICs; Africa
2	Yamey et al. (43)	31 March, 2020	Commentary	N/A	5 (Expert Opinion)	Donors' agenda & funding; market forces; rational allocation and use	International & regional	LMICs
3	Siow et al. (44)	22 April, 2020	Editorial	N/A	5 (Expert Opinion)	Affordability	Health sector	LMICs
4	Schuklenk (45)	28 April, 2020	Commentary	N/A	5 (Expert Opinion)	Availability	Health sector	LMICs; South East Asia; India; South America; Brazil; Africa; Uganda
5	Nkengasong (4)	30 April, 2020	Commentary	N/A	5 (Expert Opinion)	Market forces; accessibility; reliable health and supply systems	International & regional; Health sector	LMICs; Africa
6	Bollyky et al. (46)	7 May, 2020	Commentary	N/A	5 (Expert Opinion)	Market forces; accessibility; reliable health and supply systems	International & regional	LMICs
7	Kavanagh et al. (47)	7 May, 2020	Commentary	N/A	5 (Expert Opinion)	Accessibility; donors' agenda & funding; architecture; affordability; availability; market forces; quality; innovation	International & regional; National cross-sectoral public policy; Health sector	LMICs; South East Asia; China, Africa; Rwanda, South Africa, Malawi
8	Wong (48)	15 May, 2020	Commentary	N/A	5 (Expert Opinion)	Market forces; architecture; donor's agenda and funding	International & regional; National cross-sectoral public policy	LMICs; Asia; Thailand; Malaysia; India; Indonesia; South America; Brazil; Ecuador; Chile; Africa; Ghana; Zimbabwe; Rwanda; Zambia; Mozambique
9	Maclean and Marks (49)	17 May, 2020	News Report	N/A	N/A	Availability; accessibility; affordability; market forces	International & regional; health sector	LMICs; Africa; Central African Republic, Liberia; Senegal; Burkina Faso
10	Nature (50)	21 May, 2020	Editorial	N/A	5 (Expert Opinion)	Transparency	International & regional	LMICs
11	Chiriboga et al. (51)	30 May, 2020	Correspondence	N/A	5 (Expert Opinion)	Donors' agenda and funding; market forces; rational allocation and use; architecture	International & regional	LMICs
12	Mukwege et al. (52)	22 June, 2020	Commentary	N/A	5 (Expert Opinion)	Acceptability; architecture; reliable health and supply systems; accessibility	National cross-sectoral public policy; Health sector	Democratic Republic of Congo
13	Tangwa and Munung (53)	27 June, 2020	Commentary	N/A	5 (Expert Opinion)	Quality; safety; architecture; innovation; reliable health and supply systems	International & regional; National cross-sectoral public policy	LMICs; Africa; Madagascar; Cameroon; South Africa
14	Starr et al. (54)	3 July, 2020	Correspondence	N/A	5 (Expert Opinion)	Availability	Health sector	LMICs
15	Karim (55)	25 July, 2020	Correspondence	N/A	5 (Expert Opinion)	Affordability; accessibility	International & regional	LMICs
16	Forman et al. (56)	31 July, 2020	Commentary	N/A	5 (Expert Opinion)	Donors' agenda and funding	International & regional	LMICs
17	Moon et al. (WHO Global Preparedness Monitoring Board) (57)	20 August, 2020	Technical Report	N/A	N/A	Appropriateness; transparency; architecture; market forces; quality; affordability; donors' agenda and funding; rational allocation and use	International & regional	Global; LMICs
18	Eyawo and Viens (58)	25 August, 2020	Commentary	N/A	5 (Expert Opinion)	Market forces	International & regional	LMICs
19	Callaway (59)	27 August, 2020	News Report	N/A	N/A	Availability; market forces; transparency	International & regional	LMICs
20	Mantena et al. (60)	1 September, 2020	Commentary	N/A	5 (Expert Opinion)	Availability	Health sector	LMICs; Africa; Malawi; Uganda; Nigeria
21	Phelan et al. (61)	7 September, 2020	Commentary	N/A	5 (Expert Opinion)	Donors' agenda and funding; transparency; market forces; rational allocation and use	International & regional	LMICs
22	Bhopal and Nielsen (62)	10 September, 2020	Commentary	N/A	5 (Expert Opinion)	Transparency; acceptability	International & regional; Individual, households & community	LMICs
23	Torres et al. (63)	25 September, 2020	Commentary	N/A	5 (Expert Opinion)	Innovation; reliable health and supply systems	Health sector; International & regional	LMICs
24	Graham et al. (64)	28 September, 2020	Commentary	N/A	5 (Expert Opinion)	Availability	Health sector	LMICs; Africa; Nigeria
25	Palafox et al. (65)	28 September, 2020	Commentary	N/A	5 (Expert Opinion)	Availability	Health sector	LMICs; Philippines
26	Prabhu et al. (66)	15 October, 2020	Review Article	Scoping Review	1 (Quantitative/Qualitative or Mixed-Methods Systematic/Scoping Review)	Accessibility	Health sector	LMICs
27	Olaru et al. (67)	23 October, 2020	Commentary	N/A	5 (Expert Opinion)	Availability; rational allocation and use; appropriateness	Health sector	LMICs; Zimbabwe
28	Malpani et al. (68)	31 October, 2020	Editorial	N/A	5 (Expert Opinion)	Transparency; donors' agenda and funding; market forces	International & regional	LMICs
29	McMahon (69)	30 November, 2020	Commentary	N/A	5 (Expert Opinion)	Market forces; accessibility; transparency; architecture; donors' agenda and funding	International & regional; National cross-sectoral public policy	LMICs; Africa; South Africa; Asia; India
30	Mullard (70)	30 November, 2020	News Report	N/A	N/A	Market forces; availability; affordability	International & regional	LMICs
31	Halabi et al. (71)	3 December, 2020	Commentary	N/A	5 (Expert Opinion)	Market forces; availability	International & regional	LMICs
32	The Lancet (72)	5 December, 2020	Editorial	N/A	5 (Expert Opinion)	Availability; accessibility; affordability; market forces; donors' agenda and funding; reliable health and supply systems; sustainable financing	International & regional; health sector	LMICs; Africa; Nigeria
33	Lomazzi et al. (73)	8 December, 2020	Editorial	N/A	5 (Expert Opinion)	Donors' agenda and funding; reliable health and supply systems; sustainable financing; availability; accessibility; affordability	International & regional; health sector	LMICs
34	Dhai (74)	15 December, 2020	Editorial	N/A	5 (Expert Opinion)	Availability; accessibility; market forces; donors' agenda and funding; transparency; architecture; acceptability	International & regional; Individual, households & community	LMICs; Africa; South Africa
35	Nhamo et al. (75)	15 December, 2020	Empirical Research	Qualitative Methods	2 (Quantitative/Qualitative or Mixed-Methods Synthesis)	Affordability; accessibility; availability; market forces	National cross-sectoral public policy; Health sector; International & regional	LMICs; Brazil; South Africa
36	Schwartz (76)	15 December, 2020	Editorial	N/A	5 (Expert Opinion)	Transparency; appropriateness; reliable health and supply systems	International & regional; health sector; health service delivery	LMICs
37	So and Woo (77)	15 December, 2020	Empirical Research	Quantitative Methods	2 (Quantitative/Qualitative or Mixed-Methods Synthesis)	Availability; market forces; affordability; transparency	International & regional	LMICs
38	Wang et al. (78)	15 December, 2020	Empirical Research	Mixed Methods	2 (Quantitative/Qualitative or Mixed-Methods Synthesis)	Accessibility; availability; market forces; architecture; reliable health and supply systems; rational allocation and use; sustainable financing; affordability	International & regional; Health sector; Health service delivery; Individual, households & community	LMICs
39	Cohen and Kupferschmidt (79)	18 December, 2020	Editorial	N/A	5 (Expert Opinion)	Availability; accessibility; market forces; affordability; donor's agenda and funding	International & regional	LMICs
40	Fofana (80)	28 December, 2020	Empirical Research	Qualitative Methods	2 (Quantitative/Qualitative or Mixed-Methods Synthesis)	Reliable health and supply systems; sustainable financing	National cross-sectoral public policy; International & regional	LMICs; Africa; Kenya
41	Gostin et al. (81)	1 January, 2021	Commentary	N/A	5 (Expert Opinion)	Reliable health and supply systems; market forces; rational allocation and use; accessibility; donors' agenda & funding; architecture	International & regional; Health sector; National cross-sectoral public policy	LMICs
42	Herzog et al. (82)	5 January, 2021	Commentary	N/A	5 (Expert Opinion)	Market forces; rational allocation and use	International & regional	LMICs; Indonesia; Vietnam; Mexico; Brazil; Iran; Ecuador; Kenya; Senegal; Thailand
43	Lucerno-Prisno III et al. (83)	7 January, 2021	Commentary	N/A	5 (Expert Opinion)	Accessibility; reliable health and supply systems; appropriateness; affordability; market forces; donors' agenda and funding; availability	Health sector; International & regional	LMICs; Africa
44	Amaya and De Lombaerde (84)	9 January, 2021	Commentary	N/A	5 (Expert Opinion)	Market forces; availability; affordability; transparency	International & regional; National cross-sectoral public policy	LMICs
45	Nature (85)	14 January, 2021	Editorial	N/A	5 (Expert Opinion)	Market forces; availability; transparency	International & regional	LMICs
46	Figueroa et al. (86)	21 January, 2021	Commentary	N/A	5 (Expert Opinion)	Reliable health and supply systems; market forces	Health service delivery level; International & regional	LMICs
47	Usher (87)	23 January, 2021	News Report	N/A	N/A	Transparency; donors' agenda and funding	International and regional	LMICs
48	Paremoer et al. (88)	28 January, 2021	Commentary	N/A	5 (Expert Opinion)	Donors' agenda & funding; market forces; accessibility; affordability	International & regional; National cross-sectoral public policy; Health sector; Individual, households & community	LMICs; India
49	Koff et al. (89)	3 February, 2021	Commentary	N/A	5 (Expert Opinion)	Accessibility; market forces; affordability; reliable health and supply systems; rational allocation and use	Health sector; Health service delivery; International & regional	LMICs
50	Saleh et al. (90)	5 February, 2021	Commentary	N/A	5 (Expert Opinion)	Acceptability; availability, accessibility; reliable health and supply systems; rational allocation and use	Individual, households & community; Health sector	LMICs; Lebanon
51	Alhassan et al. (91)	11 February, 2021	Review Article	Scoping Review	1 (Quantitative/Qualitative or Mixed-Methods Systematic/Scoping Review)	Availability	Health sector	LMICs; Africa; Uganda
52	Wouters et al. (92)	12 February, 2021	Empirical Research	Mixed Methods	2 (Quantitative/Qualitative or Mixed-Methods Synthesis)	Availability; market forces; transparency; accessibility; affordability; donors' agenda & funding; sustainable financing; architecture; acceptability, reliable health and supply systems; appropriateness	International & regional; Health sector; Health service delivery; Individual, households & community; National cross-sectoral public policy	LMICs; Africa; Nigeria; Asia; Pakistan, Lebanon
53	Herlitz et al. (93)	15 February, 2021	Editorial	N/A	5 (Expert Opinion)	Rational allocation and use; reliable health and supply systems; appropriateness; transparency	International & regional; health sector; health service delivery; National cross sectoral public policy	LMICs
54	Alaran et al. (94)	17 February, 2021	Commentary	N/A	5 (Expert Opinion)	Market forces; accessibility; affordability; acceptability	International & regional; Individual, households & community	LMICs
55	Hurley (95)	25 February, 2021	Editorial	N/A	5 (Expert Opinion)	Donors' agenda and funding; market forces; availability; transparency; rational use and allocation	International & regional	LMICs
56	Yamey (96)	25 February, 2021	Commentary	N/A	5 (Expert Opinion)	Availability; market forces	International & regional	LMICs
57	Eccleston-Turner and Upton (97)	2 March, 2021	Empirical Research	Qualitative Methods	2 (Quantitative/Qualitative or Mixed-Methods Synthesis)	Market forces; donors' agenda & funding; availability; affordability; reliable health and supply systems; architecture; rational allocation and use	International & regional	LMICs
58	Bright et al. (98)	3 March, 2021	Commentary	N/A	5 (Expert Opinion)	Availability; market forces; accessibility; affordability; innovation; quality; safety	International & regional; Health sector; Individual, households & community	LMICs; Africa
59	del Castillo (99)	5 March, 2021	Correspondence	N/A	5 (Expert Opinion)	Availability; market forces	International & regional	LMICs
60	The Lancet (100)	13 March, 2021	Editorial	N/A	5 (Expert Opinion)	Market forces; availability; donors' agenda and funding	International & regional	LMICs; Africa
61	Ho and Dascalu (101)	15 March, 2021	Commentary	N/A	5 (Expert Opinion)	Accessibility; availability; market forces; affordability; acceptability; Appropriateness; adoption; rational allocation and use	International & regional; Health sector; Individual, households & community	LMICs; Eritrea; South Sudan; Haiti; South Africa
62	Shaibu et al. (102)	15 March, 2021	Commentary	N/A	5 (Expert Opinion)	Availability; rational allocation and use; reliable health and supply systems	Health sector; Health service delivery	LMICs; Africa; Kenya; Zimbabwe; Botswana
63	Guzman et al. (103)	16 March, 2021	Commentary	N/A	5 (Expert Opinion)	Market forces; affordability; transparency; donors' agenda & funding; accessibility; architecture	International & regional; National cross-sectoral public policy level	LMICs; South America; Costa Rica; Ecuador; Mexico
64	Choi (104)	18 March, 2021	Commentary	N/A	5 (Expert Opinion)	Innovation; market forces; accessibility; affordability; availability	International & regional	LMICs; Africa; South Africa; South America
65	Billette de Villemeur et al. (105)	25 March, 2021	Correspondence	N/A	5 (Expert Opinion)	Market forces; architecture	International & regional	LMICs
66	Saksena (106)	25 March, 2021	Commentary	N/A	5 (Expert Opinion)	Market forces; donors' agenda & funding; sustainable financing	International & regional; National cross-sectoral public policy	LMICs
67	World Bank (107)	30 March, 2021	Technical Report	N/A	N/A	Reliable health and supply systems; acceptability	Health service delivery; Health sector	LMICs
68	Sharun et al. (108)	31 March, 2021	Commentary	N/A	5 (Expert Opinion)	Market forces; accessibility; availability; affordability	International & regional	LMICs
69	Nature (109)	1 April, 2021	Editorial	N/A	5 (Expert Opinion)	Availability; market forces	International & regional	LMICs
70	Hussain et al. (110)	5 April, 2021	Empirical Research	Qualitative Methods	2 (Quantitative/Qualitative or Mixed-Methods Synthesis)	Availability; accessibility	Health sector; Health service delivery	LMICs; Asia; Bangladesh
71	Denhard et al. (111)	6 April, 2021	Empirical Research	Mixed Methods	2 (Quantitative/Qualitative or Mixed-Methods Synthesis)	Availability; accessibility; reliable health and supply systems; sustainable financing	Health sector; Health service delivery	LMICs; Africa; Mozambique
72	Pagliusi et al. (112)	7 April, 2021	Conference Report	N/A	5 (Expert Opinion)	Donors' agenda and funding; market forces; accessibility; availability	International & regional	LMICs

^a^*Joanna Briggs Institute (JBI) Levels of Evidence: https://jbi.global/sites/default/files/2019-05/JBI-Levels-of-evidence_2014_0.pdf*.

### Data Synthesis and Quality Assessment

One researcher (EB) applied the search strategy and screened all articles while both researchers (EB, BS) collated all the articles and reached a consensus on the finally selected papers.

## Results

### Flow Diagram of Studies Retrieved for Review

**Figure 2:**
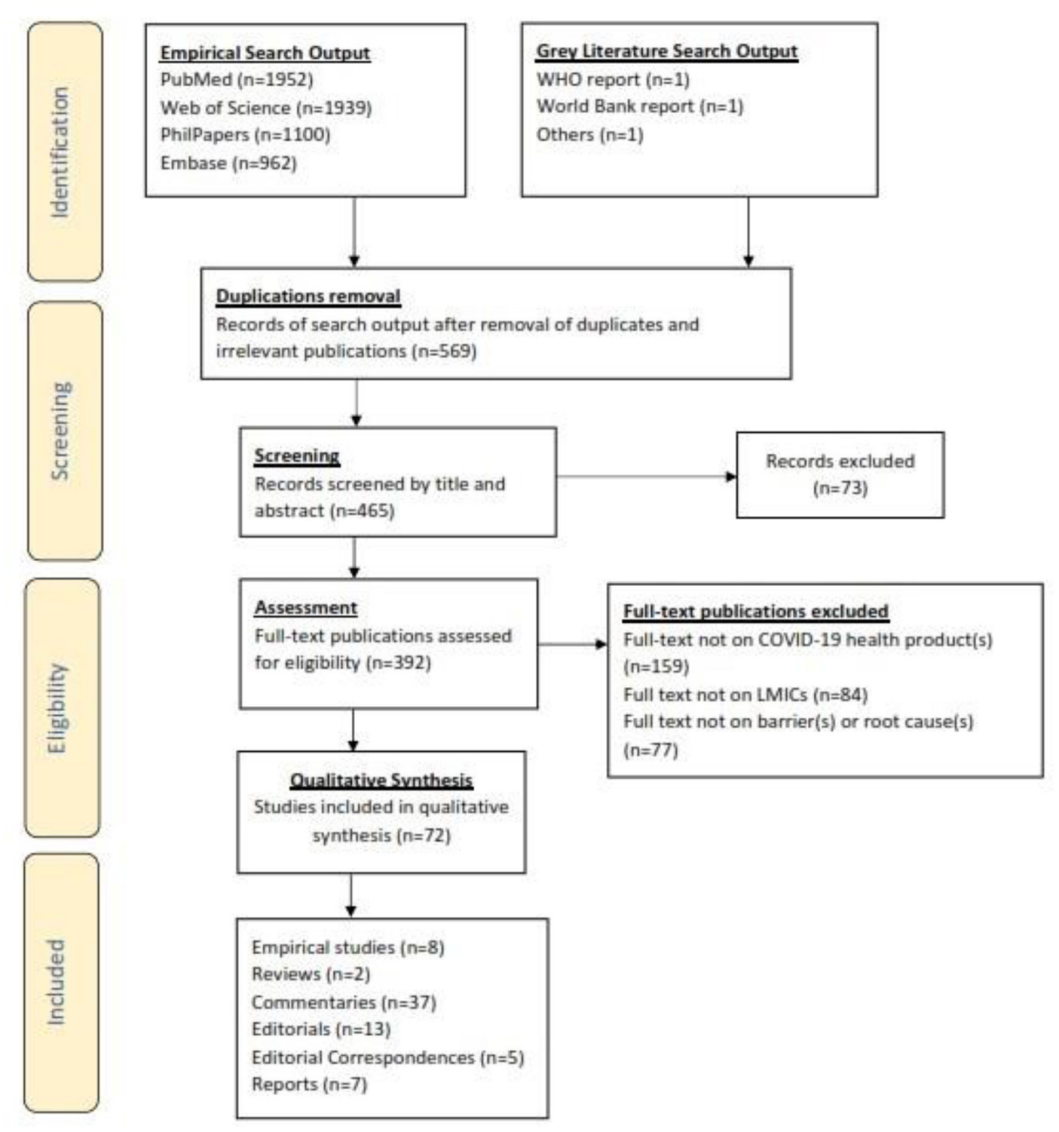
PRISMA-ScR flowchart showing how articles reporting on barriers to COVID-19 health products in low-and middle-income countries during the COVID-19 pandemic were included in the systematic review.

The PRISMA-ScR flow diagram illustrating the selection process used to include the finally selected articles is summarized in Figure 2.

### Study Selection and Characteristics

The initial search yielded 5,956 articles from database searches and purposive gray literature searches. After removal of duplicates, and screening of papers by titles, abstracts and full-text reviews, 72 publications were retained, synthesized and analyzed. They included empirical studies (*n* = 8), reviews (*n* = 2), commentaries^2^ (*n* = 37), editorials (*n* = 13), editorial correspondences (*n* = 5), and reports (*n* = 7) (More details in Table 2).

### Risk of Bias

We used a modified version of JBI's level of evidence for meaningfulness grade outline as a proxy to assess the risk of bias as outlined in Table 2. Summarily, 86.1% of the finally selected papers had a high risk of bias because they were published as expert opinion pieces with no clear research questions or methodological approach to providing evidence. The remainder of the papers (13.9%) had a low risk of bias because they had clearly stated research question and/or designs, data collection and analytical methods.

### Main Findings

All the publications shed a light on barriers to COVID-19 health products or their root cause in the context of LMICs. In terms of the geographical contextual spread, most of the articles focused on Africa (*n* = 22 or 31%). Six publications specifically shed insights on barriers to COV1D-19 health products in Asia, while three noted insights on South America. A few others were focused on countries like South Africa (*n* = 6), India (*n* = 3), Kenya (*n* = 3), Nigeria (*n* = 3), Zimbabwe (*n* = 3), and Uganda (*n* = 3).

**Figure 3:**
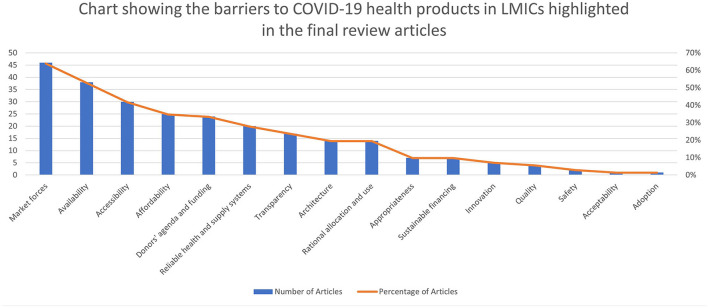
Chart showing the barriers to COVID-19 health products in LMICs highlighted in the final review articles.

**Figure 4:**
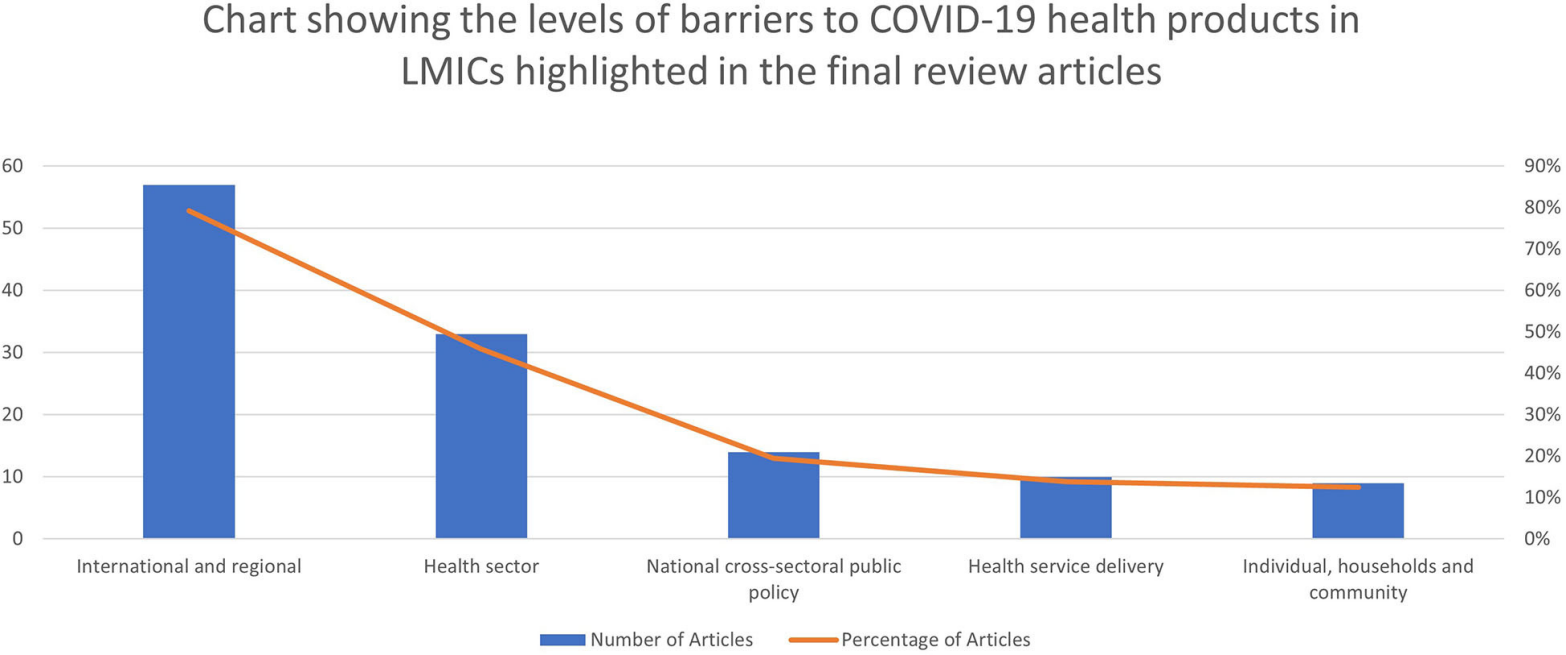
Chart showing the levels of barriers to COVID-19 health products in LMICs highlighted in the final review articles.

The five most common domains of access that caused barriers to COVID-19 health products as highlighted in the papers are market forces (64%)^3^, availability (53%), accessibility (42%), affordability (35%), donors' agenda and funding (33%) and reliable health and supply systems (28%). The three most common levels of access that caused barriers are international and regional (79%), health sectoral (46%), and national cross-sectoral [public policy] (19%). The resulting pool of evidence on the various access domains and levels that caused barriers to COVID-19 health products are summarized in Table 2 and Figures 3, 4.

## Discussion

The following is a narrative and thematic synthesis of the evidence generated on barriers to COVID-19 health products in low-and middle-income countries and the root cause of these barriers.

### Market Forces

The sudden rush and high demand for COVID-19 health products at international and regional levels at the beginning of the pandemic caused market competition and disruption of supply chains (42, 78). For instance, there was panic buying and irrational use of N95 masks by individuals and States in HICs which contributed to the shortages in LMICs (101). Global protectionism also prevailed, which is why over 70 countries imposed export restrictions of COVID-19 health products from their territories (4). For example, European and Asian countries imposed export restrictions on PPE, ventilators and other health products (46). Some HICs like the US even strove for greater efficiency in market access by mobilizing the military to oversee procurement of these health products and went as far as imposing legislative measures like the defense production act (DPA) to ban export of medical supplies and raw materials outside the US thereby forcing manufacturers to prioritize national or regional production or supply (57, 61, 75, 101). HICs were essentially outbuying LMICs in access to PPE, ventilators, vaccines, diagnostics, therapeutics, etc (58). This eventually led to restricted supply of test reagents, consumables, polymerase chain reaction (PCR) machines, and so forth to LMICs especially in Africa from competition with HICs and because various multinational health product manufacturers like Roche, Abbott, Cepheid could not quickly scale up manufacturing to meet the demand (47, 113, 114).

Market forces were at play because demand clearly outstripped supply. As of December 2020, twelve vaccine manufacturers had announced vaccine production numbers to the tune of 10 billion doses slated for 2021, but global demand (of 2-dose vaccine regimens) to achieve herd immunity (of 60–80% of worldwide population) far exceeded supply at that point (78). It is worth noting that none of the vaccine manufacturers delivered on their production projections in the early and mid-phases of the pandemic and LMICs largely suffered the brunt of the delays while other HICs sometimes received a surplus delivery of vaccines (115, 116). Further complicating all of this is the fact that the gigantic global demand for vaccines also placed a huge pressure on supply chains for starting and raw materials and other necessary components like glass vials, syringes, etc (92).

The outbuying of LMICs in access to vaccines, therapeutics has been commonly termed “vaccine/treatment nationalism” and the vaccine/treatment nationalism efforts by HICs to seek preferential access to COVID-19 health products jeopardized their access in LMICs (47, 69, 72, 73, 85, 99, 112, 117). Vaccine nationalism in particular was instigated by wealthy countries *via* APAs with manufacturers of vaccines (while they were still in clinical trials), leaving very poor countries like Mozambique scrambling for whatever is left (46, 61, 71, 83, 98, 108, 111). As early as November 2020, HICs had already reserved 51% of the 7.48B doses of vaccines that were to be produced by 13 manufacturers, though they make up about 16% of the world's population (77, 96, 106). Poor countries accounting for about 80% of the world's population had to scramble for less than one third of what's left (92, 109). For example, 80% of Pfizer's supplies had already been bought up by the US, UK, EU and Japan (84). One author indicated that 5.96B vaccine doses was the anticipated global manufacturing capacity by end of 2021, although Airfinity (as at August 2020) had estimated only 1B doses would be available by that timeline, also at odds with CEPI's earlier prediction of 2–4B doses by Q4 2021 (59, 77). The US had reserved 800M doses of six vaccine candidates, with the possibility of 1B more doses but accounted for a fifth of global COVID-19 cases; Japan, Australia and Canada combined had reserved over 1B doses though they accounted for <1% of COVID-19 cases at the time of analysis (59, 77).

Some MICs like Indonesia and Vietnam later joined the bandwagon of HICs who were making APAs with manufacturers, further hindering access for their counterparts and by February 2021, there were 62 high, middle and low-income countries or blocs that had signed APAs with manufacturers (82, 92, 103). APAs by LICs or MICs are however definitely not enough to cover their entire populations (103, 104).

The TRIPS agreement has widely been discussed in the literature as a barrier that is sustaining and exacerbating the current market imbalances (72, 84, 86, 105). The patent system is protected by TRIPS to guarantee monopolies for innovative manufacturers, but this measure, compounded by the self-interested nationalist actions of wealthy countries in a global emergency, limits the number of manufacturers and countries that can supply a product, and concentrates the manufacturer's market power with a risk for abusive pricing (57, 72, 100). For instance, with TRIPS, only a handful of multinational pharmaceutical companies and contract manufacturers can produce vaccines, thereby reducing market competition and keeping prices high. Countries and patent holders who enforce TRIPS, claim that it enables them recoup development costs and the cost of past failures, but the opaqueness of the private governance nature of TRIPS makes it hard to assess this (69).

In October 2020, India and South Africa put forth a proposal at the WTO for a waiver of the TRIPS agreement for COVID-19 medical products until the end of the pandemic (92). The original TRIPS waiver proposal, was co-sponsored and supported by more than 100 countries and territories, (including China and Russia) but faced stiff opposition by mostly HICs like the US, UK, EU countries, Norway, Switzerland, Australia, South Korea, and Japan (69). Some of these HICs like the US and EU eventually supported a waiver of only intellectual property protections for COVID-19 vaccines. After several months of intense negotiations, debate and advocacy, the WTO in its recently concluded 12th ministerial conference passed a TRIPS waiver of IP for vaccines for an initial 5 year term, with the possibility to consider a waiver for other technologies like therapeutics and diagnostics down the line. Time will tell how and if this diluted TRIPS waiver will improve vaccine production and supply in LMICs as it is generally believed to not be far-reaching enough to achieve this (118).

Many legal experts, and civil society advocates, believe the waiver waiver in its original form would have been a bold and critical move to fast track production, supply and availability of COVID-19 vaccines, diagnostics and therapeutics, especially in the mid and long term (119–121). The original proposal was also publicly supported by other social justice advocates, former and current heads of state, parliamentarians, and heads of multilateral institutions like the UN and WHO. It's important to note that the TRIPS agreement currently has measures, (commonly referred to as flexibilities) to safeguard access to health products in a national emergency at the discretion of nation states and territories, but some experts believe these measures, and the TRIPS agreement itself were never designed for use in a global health emergency or pandemic of this nature (109). Also many low resource countries currently have inadequate legislative frameworks or governance mechanism and technical capacities that allow them to institute or implement TRIPS flexibilities like compulsory licenses and parallel imports in this pandemic (69, 103, 106). Those that do, generally avoid using them to prevent retaliatory trade attacks and/or to avoid the complicated nature of imposing and implementing them (48). Opponents of the TRIPS waiver, believe intellectual property (IP) is not a barrier to scale up access to COVID-19 health tools. They place the blame at the feet of the (complex) technological know-how it takes to produce some of these products (like vaccines), and the inaccessibility or lack of capacity to produce them in LMICs (97). For example, many poor countries lacked and still lack capacity to produce PPE nationally or regionally (42). Ultimately, vaccine production is indeed very complex and so cannot easily be reverse-engineered like small drug molecules. It would require more than just waiving IP, but also sharing trade secrets, coupled with other tech know-know (106). This has proven to be very difficult because there has been a lack of commitment by multinational pharmaceutical manufacturers to transparency or the principles of open science and innovation during the pandemic (50). More on this below.

### Availability

Pre-existing the market forces constraints was a dearth of health technologies, resources and equipment for optimally responding to infectious diseases like COVID-19. China at the early onset of the epidemic only had an estimated 3.6 critical bed per 100,000 population, which was grossly insufficient (47). Many other LMICs had it far worst. There were 2.3 and 10 ICU beds per 100,000 people, in India and Brazil respectively, and in Brazil half of the beds were in the private sector (45). Malawi had less than 25 critical care beds for 19 million people or 0.1 bed per 100,000 people and Uganda had only 55 critical care beds in 12 operational ICUs for 43 million people, with 80% of them located in Kampala (45, 47, 91). Only 16% of health facilities in Kenyan counties reportedly had essential equipment to treat COVID-19, with only 22 out of 47 counties having functional ventilators in their ICUs, and in Chad and South Sudan, <10 ventilators served the entire populace (47, 60, 102). Senegal and Liberia reportedly had only 4 and 6 ventilators respectively, with one of the ventilators in Liberia being exclusively for US diplomats and citizens. In Burkina Faso, there were 11 ventilators for 20 million people while in the Central African Republic (CAR), there were 3 ventilators for a population of 5 million (49). In high-altitude geographies like Ethiopia and Bolivia, these ventilators were not only insufficient to cover the entire populations, but were also not adapted to the peculiar environmental and physiological needs in their contexts (122).

A regional WHO survey estimated that 10 African countries had no ventilators, and that <2,000 ventilators were available in public hospitals across 41 countries on the continent. There were also <5,000 intensive care beds across 43 of 55 African countries or about 5 beds per million people, although another author reported an estimated 9,800 ICU beds across Africa (49, 101). In Nigeria, the ministry of health conducted a survey revealing that only 55% of tertiary hospitals had a functional oxygen delivery system and only 11% had pulse oximeters (64). In Ethiopia, pulse oximeters were largely unavailable and in Uganda, only 38% of hospitals had oxygen, and none of the country's 16 regional referral hospitals had sufficient pulse oximeters to meet patient needs (54, 64). Only 50% of ICUs in Malawi had N95 masks and in Mozambique, only 3.4% of public health facilities surveyed had an oxygen delivery equipment, most of which were located in the Tete and Cabo Delgado provinces (60, 111).

There was also widespread unavailability of masks, gloves and other PPE in Africa early on (47). This mostly affected health workers like nurses in countries like the DRC, Kenya, Zimbabwe and Botswana where they did not have enough PPE to protect themselves nor did they have molecular tests to confirm clinical suspicions of COVID-19 (52, 102). In LMICs, like the Philippines, community health workers in addition to their regular tasks, bore the responsibilities for undertaking COVID-19 surveillance, contact tracing and monitoring individuals in quarantine and isolation, and yet many of them lacked adequate tools like PPE to do their jobs effectively and to mitigate their own risks of contracting COVID-19 (65, 88). Some health workers, like those in Bangladesh improvised to survive this ordeal by disinfecting masks for reuse, but this measure was not enough or ideal (110).

Africa accounts for only 3% of global medicinal drug manufacturing capacity whilst 70–90% of medicines consumed in sub-Saharan Africa are imported. Dexamethasone, one of the clinically proven and recommended therapeutic for treating moderate to severe COVID-19 (as at the time of writing) is not produced in Africa (98). The 12 major producers of hydroxychloroquine (no longer recommended by WHO for COVID-19 prevention/treatment) are in Asia Pacific, Europe, North Africa, Middle East and Americas; none in sub-Saharan Africa as at the time of writing (98, 123). Also, more recently approved and recommended therapeutics like tocilizumab, nirmatrelvir/ritonavir and molnupiravir are not yet manufactured in Africa, but there have been different initiatives to facilitate licensing agreements and technology transfer between originator manufacturers (like Pfizer and Merck) and generic manufacturers in LMIC regions, including Africa (124–128).

Some of the acute shortages of health tools like COVID-19 diagnostics, PPE and other consumables was due to an overwhelmingly greater demand over supply as described above (49). In war-torn settings like Libya, Syria, Yemen, contexts under dictatorship rule like Eritrea, North Korea and other fragile or politically unstable settings like Myanmar, the unavailability of health products was chronically linked to protracted wars, armed conflicts and/or civil unrests that have left these humanitarian settings isolated from global trade, tourism and sometimes international development aid in a backdrop of conditions that make the spread of infectious diseases more conducive (129–132).

The stock piling and export restrictions of critical COVID-19 health technologies by HICs and some MICs as they proved effective following clinical trials, also complicated procurement in African LMICs (51, 98). It cannot be stated enough how vaccine nationalism *via* bilateral APAs between high, middle-income countries and vaccine manufacturers undermined COVAX's success and reduced availability and affordability of vaccines in COVAX's portfolio (97). More on this to follow.

### Accessibility

At health service delivery and health sectoral levels, many LMICs are bedeviled by very poor geographical access to health products and facilities for prevention or treatment of COVID-19. For example in Mozambique, only 35% of the population had adequate access to an oxygen-ready health facility within a 1 hr drive time geographically (111).

At national cross-sectoral public policy levels, some resource poor countries hurriedly went ahead to implement lockdowns (after COVID-19 was detected within their borders) which are believed to have further exacerbated national or local geographical access barriers in these settings (47, 75). For example, Uganda's national lockdown may have hindered access to care for marginalized or stigmatized populations like migrant workers and people living with HIV/AIDS (96). In India, lockdown measures caused job losses for migrant workers, many of whom had to return back to their villages. Also, the discrimination of certain groups based on their religion, ethnicity, race, caste, gender, class, nature and conditions of their work, undermines their health and access to COVID-19 health products (73, 88).

At international and regional levels, the high regional and geographic concentration of PPE in Asia, Europe and US with top producers being China, Germany and US, left all other countries, especially LMICs at the bottom of the supply chain. It would indeed be an enormous challenge to scale up manufacturing to meet worldwide demand for these products, for example the vaccines. For instance, there was no global network of pharmaceutical manufacturers for vaccines and other health technologies before the pandemic, more so in LMICs. There was some (contract) manufacturing capacity in Argentina, Brazil, India, South Africa, Thailand, but not enough to meet peak demands.

### Affordability

Ensuring affordable and equitable access to quality-assured COVID-19 health technologies in low-and middle-income countries is no small feat. As discussed above, there was ab initio a widespread unavailability of medical tools and equipment in LMICs, but this was also because many of them did not have the resources to purchase them at the scale needed (44, 89). LMICs (where over 80% of the world's population live) can simply just not compete financially with HICs (61, 92). For instance, Brazil and South Africa waited 2 months to be able to buy reagent supplies because other HICs had bought supplies in advance as the highest bidders. Also test kits and PPE were priced very high for some LMICs hence needing to rely on HICs and philanthropic or multilateral donor funds to make purchases (75). At the onset of the pandemic, many poor countries could not afford the additional costs of ventilators or critical care beds from their limited health budgets (49). Most African countries could also not afford large scale diagnostic testing (42).

The publicly disclosed prices of vaccines for purchase have varied widely, from $6 USD per course (2 doses) for the Serum Institute of India's version of the AstraZeneca vaccine, to $39 USD per course (2 doses) of BioNTech/Pfizer's vaccine to $64-$74 USD per course of Moderna's vaccine; the later ultimately are more expensive for LMICs to purchase (70, 77, 92). The US and EU are reported to have paid an estimated $19.50 USD and $18.90 USD per dose respectively for the Pfizer/BioNTech vaccine. The US is also reported to have paid $4 USD per dose and $37 USD per dose for the AstraZeneca and Moderna vaccines respectively, while Israel is reported to have paid a higher price of $30 USD per dose for the Pfizer/BioNTech vaccine to guarantee earlier access before many other HICs (103, 104). High COVID-19 vaccine prices only increase financial burden and reduce affordability for LMICs (79). An Inter-American bank funded project estimated that Costa Rica, Ecuador and Mexico would have allocated 5 times their annual vaccination budgets to cover COVID-19 vaccinations for high-risk individuals with the prices being offered for some of the COVID-19 vaccines by HICs (103). Also, it was later found that South Africa, a MIC paid double the price for the Astra Zeneca vaccine compared to the EU's HIC bloc (104). These excessive pricing challenges will likely continue as LMICs cannot enforce price controls for COVID-19 vaccines because their high-income counterparts funding vaccine R&D and scale up (like the US) will not use price controls but rather prioritize profits, thereby limiting options to make vaccines more affordable (55). The Pfizer and Moderna vaccines will also cost more for low-resource countries to store and transport in order to maintain its ultra-cold supply chain requirements, which are additional financial barriers to their use in these settings (76, 84, 108).

Some vaccine manufacturers were planning to sell premium-priced vaccines to only wealthy patients in LMICs like Bangladesh, Brazil and India (92). Multinational vaccine manufacturers also wanted to be indemnified of all liabilities that may arise from using their products, so this was a key requirement from them before they could sign purchasing contracts with countries. But for many LMICs, offering pharmaceutical companies indemnity from vaccine liabilities due to adverse effects, was financially impossible or unconstitutional hence risking the unavailability of vaccines for them (71, 90). Also, LMICs in the middle to long term cannot produce vaccines without affordable access to global supply chains (81).

COVID-19 therapeutics like hydroxychloroquine (later found by WHO to not offer significant treatment benefits and so no longer recommended) and remdesivir were very expensive because some HICs like the US were hoarding their supplies (101). Other alternative therapies like COVID-19 convalescent plasma (CCP) was also very expensive to source and use globally, and in LMICs they particularly posed a risk for the widespread transmission of blood-borne infections like HIV because of sub-optimal health systems and infrastructure for maintaining and regulating the quality and distribution of blood products (133, 134).

At the individual, household and community level, extreme lockdown measures may have caused financial access barriers for informal sector workers due to direct or indirect economic consequences they may have suffered in low resource settings with no safety nets or universal health coverage (47).

### Donors' Agenda and Funding

Donor governments and philanthropic donors from high-income settings have also largely contributed to the access challenges that have ensued. There have been few or no strings (mechanisms to guarantee affordability and accessibility) attached to the over $12B USD of public investments from HICs, philanthropic donors and multilateral partnerships advancing R&D and access to COVID-19 health technologies (56, 57, 69, 72). Prominent philanthropic and HIC donors initially also did not support the TRIPS waiver proposal and have not mandatorily ensured manufacturers from their countries share tech know-how that could expand production and access in LMICs (68, 92).

On 4 May, 2020, an EU led virtual pledging event for the ACT-A raised 8B EUR to accelerate access to COVID-19 health tools in LMICs, but HICs were still prioritizing their sovereign access to COVID-19 vaccines by (abusively) securing APAs (47, 104). For instance, the UK had pledged £548M to COVAX as of February 2021, but had also hoarded vaccines by pre-ordering at least 400M doses, enough for ≥5 doses per citizen (95, 103, 106). Some of the pledged funding from HICs bank-rolled GAVI's COVAX facility AMC, an initiative that aimed to distribute 2 billion doses of vaccines to LMICs by end of 2021. Even though the COVAX AMC was a step in the right direction to ensure equitable distribution of vaccines to LMICs, historically AMCs have been shown to only accelerate late stage development of vaccines, and did not incentivise early-stage R&D for these products (56).

HICs, international funding agencies and multi-national pharmaceutical companies by their actions since the start of the pandemic, were not so willing to support scientific research and/or clinical testing of biomedical or indigenous herbal therapies (for curing or preventing COVID-19) in Africa, signaling a sideling of African research priorities for R&D compared to global priorities that often favor western pharmaceutical therapies and markets (53). For instance, the Africa CDC alongside the WHO cautioned against use of the Madagascar herbal tonic (COVID-Organics) and other herbal remedies from Africa considered to be unproven/untested *via* clinical trials after they were already been widely used and considered effective in some African countries (98). They however did constitute a panel of experts to evaluate the efficacy of said therapies and jointly produced a research protocol and terms of reference for conducting COVID-19 herbal medicine clinical trials (98). It's important to also note that only a very limited number of herbal clinical studies have led to licensed products in Africa (53). More on this below.

WHO's independent capacity to provide decisive leadership that forcefully addresses the access inequities in LMICs is muddled because of its heavy reliance on HICs and philanthropic donors for mandatory assessed and voluntary funding and these state and philanthropic entities most often than not make funding decisions that are aligned with expectations of powerful western multinational pharmaceutical corporations. This complicates WHO's ability to truly represent the access needs of all member states equitably (63).

### Reliable Health and Supply Systems

Unreliable health and supply systems in many LMICs have proven to be serious barriers to COVID-19 health technologies. There are currently widespread logistical barriers for use and scale up of the most efficacious (mRNA) vaccines by Pfizer/BioNTech and Moderna in (rural and hard to reach populations and areas in) some low-resource settings, like in Africa (73, 83, 86, 93, 97). The Oxford/AstraZeneca vaccine has a lower efficacy but is however less demanding for logistical supply (i.e. has less requirements for very low temperatures for storage and transportation) and is also more affordable (76, 83). Many LMICs for instance did not have registries on public health workers or total adult population prior to the pandemic and/or still lack the infrastructure for identifying all eligible health workers or adults for vaccinations (86, 107, 135). This may have led to the importation of insufficient vaccines to inoculate these demographics in low-resource settings (90). Koff et al. (89) noted that 41% of WHO member States lacked adult vaccination programs for seasonal influenza prior to the pandemic and according to Wouters et al. (92) <11% of countries in Africa and South Asia (as of 2018) reported having adult vaccination programmes and many of them may face barriers in ensuring completion of 2 dose vaccination schedules (76, 89). Prior existence of such programs may have boosted adult confidence in vaccine use and would have laid the logistical ground work for the uptake of COVID-19 vaccines (89).

A lack of or delay in producing COVID-19 vaccine deployment plans was also problematic. This is reminiscent of the H1N1 2009 influenza pandemic, where delays in producing vaccine deployment plans led to an average lag time of 261 days before some African countries could receive their first doses of influenza vaccines (97). As of December 2020, the WHO estimated a 33% readiness for roll out of COVID-19 vaccines in Africa (72). The World Bank also noted this in their COVID-19 readiness assessment report which revealed that some countries did not yet have enough trained vaccinators for delivery of COVID-19 vaccines as of February 2020. Some others had also not yet finalized the target lists of service providers and vaccine points of delivery for efficient distribution. Furthermore, 27% of LMICs surveyed had not yet prepared community engagement and social mobilization strategies to encourage people to get vaccinated. So risk and safety communication, were largely unaddressed posing serious barriers to uptake (107).

Poor regulatory expertise and technical capacities at health system levels in LMICs have also caused barriers to quality-assured health products by allowing falsified or substandard COVID-19 health products to enter the market in LMICs (57). Reliance on WHO PQP and EUL process has helped some countries to temporarily overcome this barrier, but some may lack legislative backing for reliance on WHO capacity (47, 81). Many low resource countries also lack reliable energy infrastructure or supplies, storage, delivery and waste management systems needed for scaling up access to COVID-19 vaccines, therapeutics and diagnostics (92, 93).

Centralized molecular diagnostic testing led to delayed test results and avoidable added expenses in some African countries like the DRC (4, 52, 114). Some LMICs also had insufficiently trained laboratory staff and poor specimen collection and referral networks/systems (47). Finally, there has also been mismanagement of funds and supplies for COVID-19 in some low resource settings because of poor governance systems pre COVID-19 (80).

In conflict stricken humanitarian settings like Libya, Syria, Yemen, health system infrastructure and personnel like hospitals and doctors, nurses are decimated and almost non-existent because they are regularly under armed attack by warring groups (129, 130).

### Sustainable Financing

Economic crises and contractions caused by COVID-19 will impede the ability of LMICs to sustainably fund vaccination programmes or maintain COVID-19 testing and treatment for the long haul (130). The World Bank and other multilateral development banks has reserved billions of dollars for COVID-19 vaccinations in LMICs, but this means more debt will be owed by these countries (84). These increasing debts owed by LMICs and pressure by International Monetary Fund (IMF) on borrowers to implement austerity measures undermines access to COVID-19 health technologies in LMICs (88). The G20's debt service suspension initiative hopefully will provide more fiscal space to pay back debt over much longer periods than usual but it does not cover debt owed to private creditors (92).

The ACT-A faced a $16.8B USD funding shortfall in 2021 as of June 2021 (77, 92, 136). Also, COVAX has APAs with only a few of the successful vaccines approved so far, while the remaining (still being) negotiated APAs are with vaccine candidates still in the pipeline (97). A lack of sustainable financing for ACT-A and COVAX will undermine access and will further dilute the purchasing power of COVAX and other LMIC regional procurement initiatives for vaccines and other health products (79, 103, 106). ACT-A is estimated to cost ~$38B USD over 5 years, but gains offered far exceed potential COVID-19 spread and economic losses of many trillion USDs. At least $5.7B USD will also be needed to roll out efficient vaccination programs in LMICs, and this doesn't include costs of injection materials and other consumables in a backdrop of economic downturns (72, 73). Donor countries will need to “put their money where their mouth” by fulfilling their pledges to sustainably fund this initiative (112).

### Transparency

In the early throes of the pandemic, we saw a clear lack of transparency about CEPI's and GAVI's access strategy, particularly on price negotiations for supply contracts signed with vaccine manufacturers (59, 61, 84, 87). Even supply contracts that were made available *via* freedom of information acts (FOIAs) or US Securities and Exchange Commission (SEC) filings were heavily redacted (77). Terms of partnerships or agreements between originator vaccine manufacturers from HICs and contract manufacturers in LMICs were and are also still quite unclear or shrouded in secrecy (74, 92). Secrecy of the pricing agreements further limits negotiation power of LMICs who already have limited purchasing power (85, 103). Even Gates Foundation—the biggest global health philanthropy funding some of the global public health initiatives to accelerate access to COVID-19 health products in LMICs—has not publicly shared it's access terms and conditions of agreements signed with multi-national pharmaceutical manufacturers (68). A lack of clarity on how manufacturers plan to fulfill vaccine orders, how additional challenges with vaccine programme set up and scale up beyond vaccine costs would be addressed, when and what vaccines would be distributed *via* COVAX, complicated the planning of vaccination programmes (76, 85, 93). As of February 2020, some COVAX vaccines were still in clinical trials or had not been approved for use so their supply was largely still uncertain and unguaranteed (92).

A lack of transparency or secrecy about regional or national regulatory approvals for some vaccines, were also believed to have contributed to vaccine hesitancy in LMICs (62, 76). More on this in the acceptability, appropriateness and adoption barriers outlined below.

### Rational Allocation and Use

PPE were unavailable for (patient facing) health workers in LMICs who needed them the most due to irrational allocation and use of some PPE (like full body hazmat suits, disposable gowns, face shields and goggles) by (non-patient facing) health workers like security guards (for instance in Zimbabwe) and kitchen personnel (67). Also guidance and recommendations for use of health tools like rapid diagnostic tests (RDTs) and PPE in low-resource settings have often times been developed following western-initiated and led risk assessments that do not holistically match the realities on ground in low-resource settings (67). There was clearly lack of a (legally binding) global governance ethical allocation framework for equitably distributing scarce health products like vaccines during health emergencies between (wealthy and poor) countries at the start of pandemic (46, 51, 57, 61, 75, 89). Pre-existing global governance frameworks for health emergencies like the International Health Regulations (IHR) & Pandemic Influenza Preparedness (PIP) framework (for sharing biological data and benefits from pandemic influenza pathogen research) were inapplicable or non-transferable (47). Even Global Public Private Partnerships (GPPPs) like COVAX lack the legal accountability to necessitate equitable benefit sharing. As already mentioned, global equitable access to vaccines for LMICs has been grossly undermined by HICs who are hoarding these products and multinational vaccine manufacturers profiting by asserting or protecting TRIPS and tech-know how (43, 79, 81). The existing international frameworks are insufficient or unable to completely address this barrier while nationalist and protectionist actions by some rich countries are threatening international solidarity and hampering multilateral efforts to bridge the existing access gaps. For instance, GAVI's COVAX facility currently uses WHO's allocation framework to distribute vaccines to 92 ODA-eligible and 97 self-financing countries supported by donor-funded AMC, but this framework was unsuccessful in equitably distributing 2B doses by end of 2021 (59, 97, 112). One author also noted that the first phase of COVAX's proportional allocation of COVID-19 vaccines, though better than vaccine nationalism—still prevented priority access to vaccines for some hard hit LMICs like Mexico, Brazil, Iran and Ecuador compared to other less affected countries (in terms of health and economic impact from COVID-19) like Kenya, Senegal and Thailand. WHO's proportional allocation framework ignores the special risks and needs of some LMICs and is mostly seen as an incentive or pragmatic approach to ensure HICs participated in COVAX. Some scholars have proposed the ‘fair priority model' as a more suitable alternative although it's important to note that the second phase of COVAX's allocations according to WHO will take into account the risk profiles of recipient countries (82). Summarily, the siloed and western-dominated approaches used to develop, produce and distribute some of these health technologies will definitely exclude segments of LMIC populations if they do not consider contextual factors, priorities and approaches that will determine their success or failure when implemented in low-resource settings.

Political will and international solidarity/cooperation in decision-making/agenda-setting fora to fully address this barrier has been slow, however multilateral discussions ongoing at the WHO to negotiate a pandemic treaty—if successful—could unclog this and other bottlenecks at the international and regional level (47, 74, 81, 101, 106, 137, 138). It is important to ensure that global allocation frameworks for distributing COVID-19 health products first address institutional barriers, power imbalances and historical inequities between Global North and South countries to ensure equitable distribution (106, 139). There is also a need for regional public health authorities in LMICs to develop indigenous frameworks for the ethical allocation of scarce resources (during infectious disease epidemics) that are culturally appropriate for their regions and reflect the socio-political values and realities in their contexts (74, 93, 102, 140). The Africa Union (AU), Africa Centres for Disease Control and Prevention (Africa CDC) and WHO Regional Office for Africa's regional approach to ensuring equitable access to COVID-19 health products is to be widely encouraged and their collaborative and strong regional leadership in addressing this barrier is commendable. It is hoped that they will continue to receive regional and international support and funding to successfully address this and other barriers (47, 141).

Inequitable access will particularly be challenging at the individual, households and community levels for marginalized populations of refugees, migrant and domestic workers, prison inmates who are often neglected or undocumented in poor countries and/or reside in remote locations (90).

### Innovation

Innovation barriers to COVID-19 health products were mostly noted in the regional context of the African continent. The novelty of the SARS-CoV-2 virus, first meant that most of the advanced research and development (R&D) for COVID-19 health technologies had to begin in earnest during the pandemic, leaving little or no time to prepare and respond equitably and appropriately with the right technologies (57). Also, the concentration of R&D, manufacturing and distribution technical know-how and capacity in some high and middle-income countries makes it more difficult to guarantee access to these health technologies for all countries in the world in the absence of adequate international frameworks or mechanisms (as discussed above) for governing access globally in the context of a pandemic. As already previously highlighted, there is poor R&D and production capacity for COVID-19 health products in many LMICs, especially in Africa (78, 84, 104, 117). It should however be noted that some LMICs (have) contributed to COVID-19 vaccine R&D and participated in large platform clinical trials like the RECOVERY and SOLIDARITY trials (135, 142–146). There is also substantial pharmaceutical R&D and manufacturing capacity in LMICs like India, Bangladesh, Pakistan, Vietnam, South Africa, Brazil and Cuba but the competencies in these countries and regions (especially for the R&D and production of COVID-19 health products) are still not as advanced or comparable to those in HICs (147–150). LMICs must regionally and nationally increase domestic investments in R&D and manufacturing capacity to free their territories from the shackles of technological neo-dependency on HICs and other international development funders (140, 151, 152). There has been hesitant political will and international cooperation to ensure complete technology transfer of manufacturing know-how from HICs (where capacity is mostly concentrated) to LMICs (47, 63, 73, 97). Scaling up vaccine production capabilities in Africa for instance will require more than just use of TRIPS flexibilities or a TRIPS waiver because they cannot easily be translated to vaccines, and even when possible, does not in the short term address the shortages of infrastructure, tech and regulatory capacity and knowledge for making vaccines. The WHO launched the COVID-19 Technology Access Pool (C-TAP) as a panacea for access to IP, tech know-how, etc but it is a voluntary mechanism that will require stronger global health law enforcement to become effective (81). Many multinational pharmaceutical manufacturers, HICs and private philanthropies like Gates Foundation have not joined or publicly endorsed CTAP as of the time of writing, however the US recently made a landmark announcement to share patent licenses for 11 COVID-19 vaccine, diagnostic and therapeutic technologies to WHO's CTAP (68, 92, 103, 153). It remains to be seen if this announcement will lead to further transfer of technology and manufacturing know-how and capacity.

### Acceptability, Appropriateness, and Adoption

At the wake of the pandemic, Malagasy and Cameroonian herbal therapies for COVID-19 had been alleged to be effective but implementing clinical trials of these therapies was challenging, from an ethical and regulatory standpoint, but also from a technical capacity and funding stand point thus casting doubts at international, African regional, country and local levels as to its quality and safety (53). The skepticism about these therapies have been reported by some authors as anti-African bias which could influence other forms of skepticism about western COVID-19 health products (80). For instance, only 64% of South Africans in a COVID-19 vaccine acceptance survey conducted for the World Economic Forum (WEF) said they would take the vaccine and over 30% of respondents in another survey conducted in Nigeria, Pakistan, Lebanon said they would not get vaccinated when a coronavirus vaccine became available (74, 92, 93). Skepticism or hesitancy about the vaccines were for a variety of reasons including novelty of the mRNA vaccines, speed at which the currently approved vaccines were developed sparking doubts that development of some of the vaccines did not follow regulatory due process, fear of adverse effects, fatigue and emotional detachment from a protracted outbreak, mis- and disinformation *via* conspiracy theories and rumors on social media platforms, etc (74, 92, 94). Historical or protracted distrust of governments, science, westernized health systems, experts and interventions; politicization of vaccines, and doubts about their safety and efficacy by some groups, neglect and abuse of vulnerable populations in LMICs have contributed to the current challenges of vaccine hesitancy (74, 92). The frequent ambiguity and/or dissonance of public information and scientific evidence also contributed to skepticism about some non-western based (and western based vaccines) being used for large scale vaccinations (or publicly announced to be effective) before scientific peer-review, and in some instances, details of public announcements were later amended or retracted (74, 76). Under-representation of African countries in some clinical trials for COVID-19 technologies may also have reduced its acceptability and adoption because the resulting technologies recommended after these trials are sometimes not seen as appropriate for the African people and context (101).

The haste to develop (prototype) technologies to combat infectious disease outbreaks means other important criteria (e.g., physiological and environmental considerations in high altitude populations) that make them more adaptable to LMICs are often neglected at the beginning or entirety of the response (57, 122). Non-State actors in the form of transnational youth networks have tried to address this issue for instance by collaboratively designing 3D-printable open-source PPEs, ventilators and other medical supplies that are more appropriate for use in high-altitude and other LMIC settings and State actors like government authorities in Bolivia and Colombia, instead of waiting for such tools to be approved by US, UK or EU-based stringent regulators have supported local entrepreneurs and innovators with the emergency use authorizations necessary to adopt and manufacture them in-country (154).

Vaccine hesitancy is an increasingly challenging barrier to COVID-19 health products that requires a whole of society approach to effectively deal with it. Advocacy, communication and community engagement channels need to be optimized in regionally, nationally and locally appropriate and relevant ways to re-build trust, rapidly identify communication gaps and to quell mis- and dis-information. Efficient post-marketing surveillance and indemnification of adverse effects by health authorities can also help build vaccine confidence in LMICs (92).

### Coloniality

The historical heritage of colonialism in LMICs is mostly attributed to be the root cause of the barriers described above. Persistent hegemonic neo-colonial ideologies, attitudes and practices in how global health and trade is valued, governed and funded today continue to entrench these barriers and access inequities (80, 155, 156). For instance, vaccine nationalism (which has mostly been created by countries which are former colonizers) has imperialist undertones linked to historical imbalances in power and resources that have shaped the world of the haves and haves not today (94, 157). As COVID-19 has staunchly revealed, many LMICs lack the geopolitical power in global governance fora like the WTO to effectively counter neo-colonial international patent restrictions for developing or producing COVID-19 health technologies (101, 139, 156).

COVID-19 health products have commonly been referred to as global public goods (GPG) when advocating for their global access, however the neoliberal implication of GPG makes its use inherently flawed, and in the absence of clear global unanimous normative guidelines on how to consistently determine what GPGs are, there is risk of philanthropic donors and HICs determining what GPGs are to be prioritized based on subjective or non-inclusive geo-political governance processes (106). Inherent in the nature and neoliberal implication of GPG is its non-consideration of distributional equity and impact when using tools like cost effectiveness analysis and health technology assessments to determine its effects or externalities (106). In a neoliberal capitalist free market system, the public goods theory enables the government to correct market failures by private companies lacking incentives to provide certain goods. The government's role is to address the failures, not as the primary provider of the goods, hence the capitalist conception of the “public good” limits the state to being a buyer, not a producer of the good. This does not end up solving the structural issues that created the market failures and access barriers in the first place. The framing of GPGs in its newest iteration (as seen with the COVID-19 pandemic) is to maximize positive externalities that accrue from states publicly providing private goods that are deemed critically important for global health (139). These positive externalities could include reduction of deaths and cases and herd immunity from vaccines in arms which also increase the human capital available for a re-boot of the economy. But it could also include, protection of multi-national pharmaceutical manufacturers (who profiteer from the pandemic) and financial markets in HICs (106).

### Study Limitations

This review included commentaries, editorials, editorial correspondences and gray literature that often do not disclose or follow research methodologies that are used or applied in global health, and hence are generally regarded to provide the least level of meaningful evidence. This is due to the novel nature of the COVID-19 pandemic and a lack of empirical research on barriers to COVID-19 health products in low-and middle income countries. The lack of empirical research may—at least in part—be caused by the lengthy peer-reviewed publication process which is not conducive to a dynamic and rapidly changing situation.

There is also the risk that the non-external screening, quality appraisal and selection of final articles by the authors may have introduced some selection or reporting bias and/or undermined the reliability and validity of the authors' methodology and findings. Inclusion of only publications written in English may have also limited the availability of publications relevant to this review. Any gaps identified by the reader may have been due to bias, oversight, or a lack of academic interest by the authors of the papers included in this review. These gaps merit further academic investigation and some of these limitations could be addressed by conducting empirical research that will generate more insightful and actionable evidence for global health policy and/or decision makers and practitioners during the COVID-19 pandemic.

### Implication for Research and Policy

COVID-19 variants of concern and variants of interest continue to circulate globally and the ebbs and flows of COVID-19 cases suggests that seasonal waves of transmission will persist for the foreseeable future as COVID-19 becomes an endemic respiratory illness worldwide. The COVID-19 pandemic will not be the last pandemic or epidemic in our lifetime. As of 29 April 2022, the WHO is tracking more than 20 outbreaks of infectious diseases like monkey pox, middle east respiratory syndrome coronavirus (MERS-CoV), avian influenza, lassa fever and dengue that are occurring globally, but mostly in low-and middle-income countries (158). Many LMICs will need to fortify their biomedical arsenals with health technologies necessary to tackle the ever present threats of emerging infectious disease outbreaks, epidemics or pandemics. Also, access to COVID-19 vaccines, therapeutics and diagnostics remain abysmally low in Africa despite improved access in other low-resource settings in Asia and South America and despite improvements in manufacturing capacity and supply globally (159–161). Access to health tools for addressing other currently ongoing infectious disease outbreaks in LMICs is also very poor and over half of the priority diseases in WHO's R&D blueprint have no diagnostics, vaccines or therapeutics (160, 162).

This review will hopefully support policymakers at global governance, regional and national LMIC levels with the evidence needed to holistically understand and tackle the bottlenecks that continue to impede access to health products for fighting emerging infectious diseases (of pandemic potential) in low-resource settings. We hope this paper will also spur or contribute to more empirical research that could further illuminate our knowledge and understanding of the barriers to health products that are unpacked in this review.

## Conclusion

This review has outlined and elaborated on the various barriers to health products that must be comprehensively addressed to mount a successful global, regional, national and subnational response to present and future epidemics and pandemics in LMICs. Global public health authorities and international development donor and recipient country actors have had several opportunities (during previous infectious disease outbreaks) in the last few years to tackle some of the barriers noted in this review. The COVID-19 pandemic is a watershed moment for global health development actors to move beyond rhetoric to long-lasting reforms and actions that ensure global equitable access to health products during infectious disease epidemics, especially in low-resource settings.

## Data Availability Statement

The original contributions presented in the study are included in the article/Supplementary Material, further inquiries can be directed to the corresponding author/s.

## Author Contributions

EB was responsible for literature searches, data analysis and synthesis, references, and submission and acting as the corresponding author, had the final responsibility for the decision to submit for publication and wrote the first draft of the manuscript. BS contributed to the conceptualization in conversations and helped to refine the arguments and manuscript, also contributed to the data analysis, synthesis, and final manuscript writing as a supervisory senior author and assisted with proofreading and copy editing. Both authors contributed to the article and approved the submitted version.

## Author Disclaimer

The views expressed in this research paper are primarily those of the authors and do not necessarily represent the views of their affiliated institutions.

## Conflict of Interest

The authors declare that the research was conducted in the absence of any commercial or financial relationships that could be construed as a potential conflict of interest.

## Publisher's Note

All claims expressed in this article are solely those of the authors and do not necessarily represent those of their affiliated organizations, or those of the publisher, the editors and the reviewers. Any product that may be evaluated in this article, or claim that may be made by its manufacturer, is not guaranteed or endorsed by the publisher.

## Footnotes

^1^These domains of access are not homogenous in their meaning or attributes and may thus interact in multiple ways across the various levels of access. This attests to the adaptive complexity of their expression and meaning in practice.

^2^Commentary is used generally to refer to expert opinion pieces (including perspectives, viewpoints, etc) published in research journals by multidisciplinary global public health experts.

^3^The percentage is calculated based on the number of publications that discuss each access domain or level.
