# Supplementary material for: Barriers to COVID-19 Health Products in Low-and Middle-Income Countries During the COVID-19 Pandemic: A Rapid Systematic Review and Evidence Synthesis
Source: Front Public Health. 2022 Jul 22;10:928065. doi: 10.3389/fpubh.2022.928065 (PMC9354133; doi:10.3389/fpubh.2022.928065)
Supplement: Supplementary file 1 [file Data_Sheet_1.docx]

Supplementary Material

# Supplementary Tables

## Supplementary Table 1: Search algorithm applied to bibliographic databases to retrieve evidence on scoping or systematic reviews on barriers to COVID-19 health products for people living in low-and middle-income countries during the COVID-19 pandemic. The algorithm was applied as best as possible to PubMed, Cochrane and Google Scholar.

| Set | Terms |
| --- | --- |
| 1 | “review” or “scoping review” or “rapid review” or “systematic review” or “literature review” or “mapping review” or scoping study” |
| 2 | “COVID-19” or “2019 novel coronavirus” or “2019-nCoV”or “SARS-CoV*” |
| 3 | “low and middle income country*” or “low resource setting*” or “resource poor setting*” or “low income country*” or “middle income country*” or “resource constrained setting*” |
| 4 | “barrier*” |
| 5 | “access to medicine*” or “access*” |
| 6 | “global health” or “public health” or “health*” |
| 7 | “equity*” or “inequity*” |
| 8 | 2 and 3 and 4 |
| 9 | 5 and 6 and 7 |
| 10 | 1 and 8 |
| 11 | 1 and 9 |

## Supplementary Table 2: Search algorithm applied to bibliographic databases to retrieve evidence on barriers to COVID-19 health products for people living in low-and middle-income countries during the COVID-19 pandemic. The algorithm was applied to Web of Science, PubMed, Embase and PhilPapers.

| Set | Terms |
| --- | --- |
| 1 | “COVID-19” or “2019 novel coronavirus” or “2019-nCoV”or “SARS-CoV*” |
| 2 | “drug*” or “medicine*” or “therapeutic*” or “treatment*” or “vaccine*” or “diagnostic*” or “health technology*” or “health tool*” or “health product*” or “medical technology*” or “medical tool*” or “medical product*” |
| 3 | “low and middle income country*” or “low resource setting*” or “resource poor setting*” or “low income country*” or “middle income country*” or “resource constrained setting*” |
| 4 | “barrier*” |
| 5 | “access to medicine*” or “access*” |
| 6 | “global health” or “public health” or “health*” |
| 7 | “equity*” or “inequity*” |
| 8 | 1 and 2 and 3 |
| 9 | 4 and 5 and 6 and 7 |
| 10 | 8 or 9 |

## Supplementary Table 3: PCC Criteria Checklist

| Item | Description |
| --- | --- |
| Population | People living in low-and middle-income countries/low-resource settings |
| Concept(s) | Barriers to COVID-19 health products based on at least 1 of 5 levels of access and at least 1 of 16 domains of access adapted from *Bigdeli et al.* (2012). Levels of access are i) international & regional level, ii) national cross-sectoral public policy level, iii) health sector level, iv) health service delivery level and v) individual, households and community level. Domains/dimensions of access are: i) acceptability, ii) accessibility, iii) adoption, iv) affordability, v) appropriateness, vi) architecture, vii) availability, viii) donors’ agenda & funding, ix) innovation, x) market forces, xi) quality, xii) rational allocation and use, xiii) reliable health & supply systems, xiv) safety, xv) sustainable financing and xvi) transparency |
|  | Article must focus on barriers to COVID-19 health products in the framework of global health equity or inequity as described by Wernli *et al.* (2016) |
| Context(s) | COVID-19 pandemic and health products (drugs, diagnostics, vaccines, etc) in low-and middle-income countries/low resource settings |

## Supplementary Table 4: Acronyms and Abbreviations

| **ACT-A** | **Access to Covid-19 Tools Accelerator** |
| --- | --- |
| **Africa CDC** | **Africa Centres for Disease Control and Prevention** |
| **APA** | **Advance Purchase Agreement** |
| **AMC** | **Advance Market Commitment** |
| **BIPOC** | **Black Indigenous and People Of Colour** |
| **CEPI** | **Coalition for Epidemic Preparedness Innovations** |
| **DRC** | **Democratic Republic of Congo** |
| **EU** | **European Union** |
| **EUL** | **WHO Emergency Use Listing** |
| **GAVI** | **Gavi, the Vaccine Alliance** |
| **HICs** | **High Income Countries** |
| **ICU** | **Intensive Care Unit** |
| **LMICs** | **Low and Middle Income Countries** |
| **ODA** | **Official Development Assistance** |
| **PHEIC** | **Public Health Emergency of International Concern** |
| **PPE** | **Personal Protective Equipment** |
| **PQP** | **WHO Pre-Qualification of Medicines Programme** |
| **R&D** | **Research and Development** |
| **SARS** | **Severe Acute Respiratory Syndrome** |
| **TRIPS** | **Trade Related aspects of Intellectual Property rights** |
| **UK** | **United Kingdom** |
| **USD** | **United States Dollar** |
| **WASH** | **Water Sanitation and Hygiene** |
| **WHO** | **World Health Organization** |
